# A multicenter, randomized, double-blind, placebo-controlled trial evaluating the efficacy and safety of Huangqi Guizhi Wuwutang granule in patients with rheumatoid arthritis

**DOI:** 10.1097/MD.0000000000014888

**Published:** 2019-03-15

**Authors:** Yiru Wang, Yang Liu, Zhijie Xi, Yang Yu, Li Liu, Jianchun Mao, Lianbo Xiao, Xiaohua Gu, Min Yao, Xuejun Cui, Qi Shi, Yongjun Wang, Qianqian Liang

**Affiliations:** a Longhua Hospital, Shanghai University of Traditional Chinese Medicine; b Institute of Spine, Shanghai University of Traditional Chinese Medicine, 725 South Wan-Ping Road; c Key Laboratory of Theory and Therapy of Muscles and Bones, Ministry of Education Shanghai University of Traditional Chinese Medicine; d Guanghua Hospital of Integrated Traditional Chinese and Western Medicine; e Shanghai Seventh People's Hospital, 358 Gaoqiao Datong Road, Pudong New Area; f Rehabilitation Medicine College, Shanghai University of Traditional Chinese Medicine, 1200 Cai Lun Road, Shanghai, China.

**Keywords:** active rheumatoid arthritis, Huangqi Guizhi Wuwutang granule, multicenter, placebo, randomized controlled trial, traditional Chinese medicine

## Abstract

**Background:**

Rheumatoid arthritis (RA) is a chronic autoimmune disease characterized by swelling, pain, and synovial damage. Effective methods lack in the treatment of RA. A traditional prescription in use for thousands of years in China, Huangqi Guizhi Wuwutang granule (HGWG) is still chosen to relieve pain and prevent joint malformation in RA patients. However, no evidence-based medical research has been organized to assess the effectiveness and safety of HGWG for RA.

**Methods/design:**

We will conduct a multicenter, randomized, double-blind, placebo-controlled clinical trial to determine whether HGWG can relieve pain and protect joints. We will randomly divide 120 patients with active RA into 2 groups, treated for 12 weeks. Main measurement is the rate of ACR50 score (American College of Rheumatology) from the baseline to 12 weeks. Secondary measurements include rate of ACR20/70, change of Disease Activity Score (DAS) 28, Health Assessment Questionnaire-Disability Index (HAQ-DI), Patient Assessment of Arthritis Pain, Patient Global Assessment of Arthritis, and AIS score. The time points are set as baseline, 2 weeks, 4 weeks, 8 weeks, 12 weeks, 24 weeks, and 48 weeks. In addition, the rate of ACR50 from the baseline to 2 weeks, 4 weeks, 8 weeks, 24 weeks, and 48 weeks’ follow-up are also the secondary outcome measures.

**Discussion:**

The findings of this research will elucidate the efficacy and safety of HGWG and provide an alternative treatment for RA. In addition, our data will benefit the clinical decision-making on active RA and possibly be incorporated into future guidelines.

**Trial registration:**

ClinicalTrials.gov ID: NCT03593837.

## 1 Introduction

Rheumatoid arthritis (RA), a chronic systemic inflammatory disease, mainly involves the synovium, leading to joint swelling, pain, bone erosion, and even deformity directly after over-accumulation of synovial fluid and fibrous tissues.^[1]^ Its overall prevalence is lower than 1%, and annual incidence is about 0.05%.^[2,3]^ Currently, the interventional efforts for RA focus on reducing the degree of disease activity, improving physical function and life quality, and restraining the development of complications.^[4]^ However, their outcomes are still unsatisfactory. So far, safe and effective anti-RA drugs have not yet been invented.^[5]^

As a complementary and alternative medicine (CAM), Chinese herbal medicine may have the potential of remitting RA symptoms or lowering disease activity.^[6,7]^ HGWG, a traditional Chinese medicinal formula with a history of thousands of years. To safeguard its quality for a longtime, Huangqi Guizhi Wuwutang decoction can be extracted into HGWG that has been used in our hospital for decades.

Although HGWG have been used clinically for many years, the efficacy and safety of HGWG still need evidence-based medical research. So, we plan to conduct a randomized, double-blind, placebo-controlled trial to confirm the efficacy and safety of HGWG in fighting against RA.

## 2 Methods/design

### 2.1 Study design

**Figure 1:**
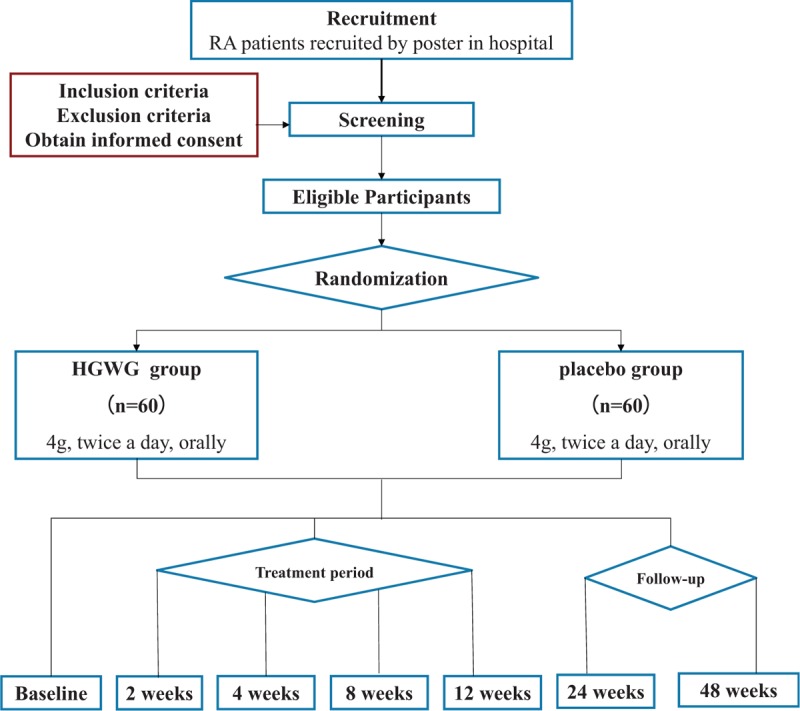
Project overview. HGWG = Huangqi Guizhi Wuwutang granule, RA = rheumatoid arthritis.

This study is a multicenter, randomized, double-blind, placebo-controlled clinical trial with 2 parallel arms (Fig. 1). The purpose of this study is to evaluate the efficacy and safety of HGWG in the treatment of RA. We will recruit 120 patients from 3 medical institutions in Shanghai, China: Longhua Hospital Affiliated with Shanghai University of Traditional Chinese Medicine, Shanghai Seventh People's Hospital, and Shanghai Guanghua Hospital of Integrated Traditional Chinese and Western Medicine. All patients will be randomly assigned to HGWG group (experimental group) and placebo (control) group.

Patients in the experimental group will be required to take in HGWG: twice a day, 1 package a time, after breakfast and dinner, for 12 weeks. Patients in the control group will use HGWG placebo in the same manner.

### 2.2 Medication and administration

HGWG will be manufactured, packaged and labeled by a factory in Shanghai based on standards for good manufacturing practice. The procedures in the making of HGWG are listed in Table 1. The crude herbs include: *Astragalus membranaceus* (Huang Qi), 9 kg; *Cassia* twig (Gui Zhi), 9 kg; *Paeonia lactiflora*pall (Shao Yao), 9 kg; *Radix Glycyrrhizae* (Gan Cao), Ziziphus zizyphus (Da Zao), 18 kg. All materials will be supplied in 1 batch from Long Hua hospital and stored in a cool and dry place. The procedures in the making of HGWG are as follows:

1. three rounds of extractions – firstly, put the herbs listed above in a ceramic container, then add 1000 L of distilled water into the container to macerate the herbs for 1 hour, and then boil the mixture at 100°C for 1 hour for the first extraction; secondly, pour out the liquid extract, and add 1000 L of distilled water and boil it at 100°C for 1 hour; thirdly, extract after adding 500 L of distilled water and boiling for half an hour.
2. concentration: concentrate the mixture of 3 extractions at 60°C (660 mm Hg) and spray-dry the mixture into powders. Then the powders are smashed and screened through a mesh size of 80. At last, the granules are packaged (4 g a bag) and stored in a clean room at 20°C and 50% humidity. HGWG placebo is made up of HGWG extraction (10%) and bitterant (90%). Pigment (such as lemon yellow, caramel pigment, and sunset yellow), edible lactose essence, and starch which makes the color, taste/smell, and shape are similar to HGWG.

**Table 1:**
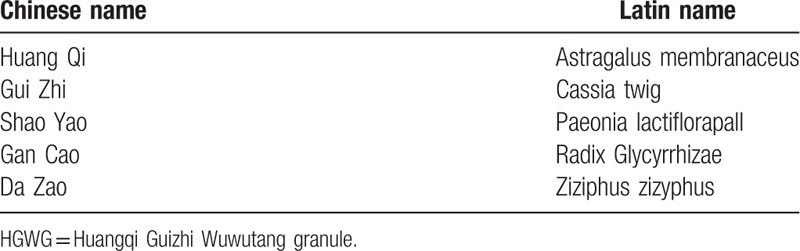
Components of HGWG.

Anti-rheumatic drugs (DMARDs), such as methotrexate (MTX) and sulfasalazine, may be used during the observation period. Patients’ original records are recorded. Non-steroidal anti-inflammatory drugs (NSAIDs) may be used to control patient's pain. The doctor responsible for the project will keep all medication records during the patients’ treatment. In addition, other conditions and treatments should be documented in the management manual. Both groups will have a 3-month treatment and a 9-month follow-up. At 7 time points (baseline, 2 weeks, 4 weeks, 8 weeks, 12 weeks, 24 weeks, and 48 weeks), visits will be scheduled for each patient. The plan is shown in Table 2.

**Table 2:**
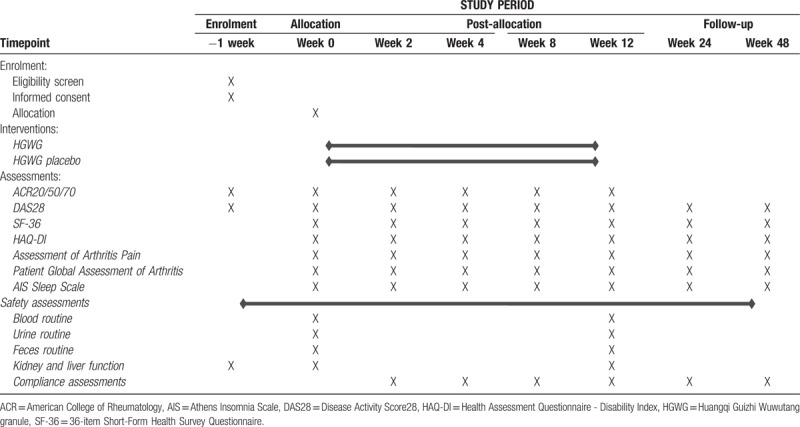
Schedule of enrollment and assessments.

### 2.3 Ethical issues

The trial will be conducted in accordance with the *Declaration of Helsinki* and Ethical Guidelines for Clinical Research, and the trial protocol has been approved by the Research Ethical Committee of Longhua Hospital Affiliated with Shanghai University of Traditional Chinese Medicine, Shanghai, China, (approval number: 2018LCSY024), Guanghua Hospital of Integrated Traditional Chinese and Western Medicine (approval number: 2018-K-10), and Shanghai Seventh People's Hospital (approval number: 2018-IRBQY-014).

The physicians will invite the patient to participate in the trial, tell them in detail why we should take this trial and what kind of rights, obligations, and risks they will have if they participate the trials. And the care provider will give them a written informed consent. Only the patients who fully understand and sign the informed consent, can participant the trial.

Additionally, the personal information about the potential and enrolled participants will be collected to be used only in this trial, and we will not share or maintained the personal information when unnecessary.

### 2.4 Participants

Recruitment notice for research participants is publicized on posters and websites at 3 hospitals (Longhua Hospital Affiliated with Shanghai University of Traditional Chinese Medicine, Shanghai Seventh People's Hospital, Shanghai Guanghua Hospital of Integrated Traditional Chinese and Western Medicine). All participants will receive informed consent before randomization.

### 2.5 Sample size calculation

We calculated the sample size according to our primary study which conducted from January 2018 to October 2018 into the efficacy of the HGWG versus HGWG placebo, and we found that patients taking HGWG achieved 83.5% ACR50, whereas patients in the placebo group achieved 54.7% ACR50 response. Formula of sample size calculation is . In this formula, N1 and N2 are the number of HGWG and placebo group, *u*_α/2_ = 1.96 when type 1 error is 0.05; *u*_β_ = 1.282 when type II error is 0.1 in two-sided tests. is the mean of *p*_1_ and *p*_2_.^[8]^ It was estimated that approximately 50 participants per group were needed to achieve 90% power and a (2-sided) 5% significance level in detecting treatment differences. We estimate that 120 patients (60 in each group) will be enrolled to ensure that the results are statistically significant, considering a reduction rate of 20%.

## 3 Criteria

### 3.1 Inclusion criteria

Participants meeting the following requirement will be included.

- Diagnosed with RA according to the diagnostic criteria developed by the American College of Rheumatology in 1987.
- DAS28 score of 2.6 to 5.1.
- Aged 18 to 70.
- Signing informed consent and ensuring test compliance.
- Women of childbearing age must undergo testing and have no pregnancy plan throughout the trial.
- Having no history of combined disease and active tuberculosis.

### 3.2 Exclusion criteria

- Having any concurrent rheumatic disease, such as systemic lupus erythematosus, Sjogren's syndrome, or severe osteoarthritis.
- Having tumor and cancer.
- Having a history of serious allergic reactions.
- Pregnant or lactating women and women of childbearing age who do not have an effective contraceptive method.
- Having cardiac, hematologic, respiratory, neurological, endocrine, renal, hepatic, gastrointestinal, or psychotic disease.
- Having infectious diseases.
- Showing poor compliance, and unwillingness to be followed-up.
- Showing alcoholism or drug dependency.

### 3.3 Interventions

Experimental group (HGWG group): Patients are given HGWG (orally twice a day for 12 weeks and instructed to dissolve one package (4 g) with hot water (200 mg). Control group: HGWG placebo will be used in the same way.

Supportive therapy of two groups:

1. DMARDs can be used during the observation period, such as MTX and sulfasalazine (the original dose will be maintained);
2. According to the patient's pain severity, NSAIDs can be added;
3. Patients should provide a detailed list of drugs used during follow-up and observation period. Researchers must record the name of the drugs, dosage, frequency, and duration.

### 3.4 Randomization and allocation

Shanghai Medical Clinical Research Center (CRC) will control the randomization develop a random number table by SAS9.1 PROC PLAN. The random number table will be generated in a ratio of 1: 1 using 3 test centers as a stratification factor. This table contains 2 corresponding groups, one is sequence number of inclusion, the other one is the treatment or placebo group.

Pharmaceutical factory will contact the CRC to obtain the random number table, then post the sequence number to boxed of medicine (HGWG or placebo) according to the table.

When a participant is recruited in, the investigator will make a central telephone call and give him/her the medicine (HGWG or the placebo) from the administrator according to the central call.

All the investigators except for the CRC and pharmaceutical factory will not know the corresponding relations between sequence numbers and different groups until the trials completed.

### 3.5 Blinding

In this trial, all the researchers will not be in contact with the CRC and the pharmaceutical factory. The CRC will be isolated from all researchers. The stuff in the pharmaceutical factory does not participant in the other parts of this trial. Therefore, the investigator, doctors, nurses, statisticians (analyze outcomes), and other participants have no access to the research information, and will not know the corresponding relations between sequence numbers and different groups until the whole trial completed (including the statistical work).

If an adverse event is claimed, we will record it and provide a proper treatment strategy. Clinicians will provide emergency services in case of serious adverse events, and report them to the Institutional Review Board within 24 hours. Then the leader of Longhua Hospital ethic committee, CRC and the main investigator will make a decision to break the blinding or not, and the participant information of intervention, HGWG or HGWG placebo will be provided by CRC.

## 4 Outcome measures

### 4.1 Primary outcome measure

ACR50 from baseline to 12 weeks after treatment will be compared. ACR50 is a standard to describe RA symptoms.^[9]^ ACR 50 is met when the number of tender joints reduces by ≥50%, the number of swollen joints by ≥50%, and at least 3 of the following 5 indexes improves by ≥50%:

- Patient's assessment on arthritis pain using a Visual Analogue Scale (VAS) of 0 to 100 mm
- Patient's global assessment of disease activity using VAS scale (0–10)
- Patient's assessment of physical function and disability HAQ-DI
- Erythrocyte sedimentation rate (ESR)
- C-reactive Protein (CRP) level

Similarly, ACR20 is met when ≥20% is achieved in the reduction of tender joint number, swollen joint number, and indexes of 3 of the 5 other measurements.^[10]^

ACR scale has been accepted as the efficacy benchmark in RA clinical trials and has greater discriminant capacity to distinguish patients on active treatment from placebo control.^[9,11,12]^ Here we choose ACR50 as the primary index for that ACR50 can provide more useful information than ACR 20/70.^[13,14]^

### 4.2 Secondary measurements

The secondary outcomes will be to compare the rates of change in the ACR20/70, DAS28, HAQ-DI, Patient Assessment of Arthritis Pain, Patient Global Assessment of Arthritis, and AIS score from baseline to 2 weeks, 4 weeks, 8 weeks, 12 weeks, 24 weeks’ and 48 weeks’ follow-up. The rate of change in the ACR50 from the baseline to 2 weeks, 2 weeks, 4 weeks, 8 weeks, 12 weeks, 24 weeks’ and 48 weeks’ follow-up are also secondary outcome measures. Additionally, the change in score on the 36-item Short-Form Health Survey Questionnaire (SF-36) from baseline to 4 weeks, 8 weeks, 24 weeks, and 48 weeks is also calculated.

The DAS28 is an index calculating the number of painful and swollen joints (28 joints, i.e. shoulders, elbows, wrists, metacarpophalangeal and proximal interphalangeal joints, and knees), ESR and the score of global assessment.^[15]^ The formula of DAS 28 is 0.56 × √(28 painful joint count) + 0.28 × √(28 swollen joint count) + 0.70 × (ln ESR) + 0.014 × GH. ESR refers to ESR. GH is the patient's general health visual analog scale (0–10 mm).^[16–18]^

HAQ-DI is a subscale of ACR 20/50/70 that reports biochemical and physical conditions. It has been widely used in RA clinical trials to assess disease activity and patient disability.^[19]^ The HAQ-DI measure shave eight dimensions of functional activity: pruning, dressing, rising, eating, walking, personal hygiene, reach, grip, and other routine activities. Each item has 4 degrees ranging from 0 to 3. “0” refers to “no functional difficulty”, “1” to a bit of functional difficulty, “2” to very much functional difficulty, and “3” to no ability to work. HAQ-DI score 0 to 1 means mild to moderate functional difficulty; 1 to 2 means moderate to severe disability; and 2 to 3 means generally severe disability.^[20]^

People with RA often have sleep disorders that worsen as RA deteriorates.^[10,21]^ The Athens Insomnia Scale (AIS) can help patients quantitatively self-evaluate sleep disorders with a psychometric instrument that includes eight indicators for sleep induction, awakenings during the night, final awakening, total sleep duration, sleep quality during the night, wellbeing during the night, functioning capacity during the day time, and sleepiness during the day time. The score for each indicator ranges from 0 to 3, and the total score is 24 points. The higher the score, the worse the sleep quality, and vice versa.^[22]^

The SF-36 measures eight dimensions, including vitality, body function, body aches, general health perceptions, body functions, emotional function, social function, and mental health. SF-36 is widely used in the evaluation of quality of life in patients with RA.^[10,23]^

In addition, concomitant medications are also recorded as secondary outcomes.

### 4.3 Safety assessments

The HGWG has been used for hundreds of years in China, and the quantity of herbs in the HGWG are safe according to the recommended amount in Pharmacopoeia of the People's Republic of China (2015 version). The incidence and severity of adverse reactions are observed in each test. Clinical tests on joint swelling, tenderness, morning stiffness, and average grip strength will be observed closely along the whole trial. Also, the laboratory tests, blood routine, urine routine, feces routine, kidney, and liver function will be performed at the beginning and end of this trial.

### 4.4 Participant timeline

The participants will be researched for 24 months, from 1st April 2019 to 1st April 2021. The final visit of all participants is scheduled for 31st July, 2021. The recruitment process is shown in Figure 1, and the schedule is shown in Table 2.

### 4.5 Data collection and monitoring

This is a 48 weeks’ clinical trial in which participants will receive 12 weeks of medication and 36 weeks of follow-up. Disease activity will be assessed at 7 time points (baseline, 2 weeks, 4 weeks, 8 weeks, 12 weeks, 24 weeks and 48 weeks). Longhua Hospital affiliated with Shanghai University of Traditional Chinese Medicine (http://www.longhua.net/ywsy/gzlc/287.jhtml) is responsible for quality control and training for investigators.

Epidata (version 3.0) procedure will be used to restrict data values. Two trained investigators will enter and compare the data independently to rule out the difference.

### 4.6 Statistical analysis

Trained statisticians will adopt intention-to-treat approach for effectiveness and safety analysis. In addition, the last-observation-carried-forward method will be applied in handling missing values. All statistical analysis will be conducted by Statistical Packages of Social Sciences (SPSS) software (version 22.0). Statistical test is two-sided and *P* < .05 is statistically significant. The mean ± standard deviation will be used for the description of continuous variables. The percentage is used to categorize the variable's description. Continuous variables following the normal distribution will be analyzed by Student *t* test; otherwise, non-parametric tests will be used to compare group differences.

## 5 Discussion

RA may lead to complications such as severe infection and malignant tumor.^[24–26]^ Inappropriately treating RA brings back with irreversible joint deformity and disability, which seriously affects the patients’ working ability and life quality of life. Doctors used DMARDs and MTX to treat RA patients with a good improvement. However, in MTX-resistant patients, the effective rate is only 25% to 40%.^[8,27–29]^ Therefore, it is urgent to develop new effective therapies. Chinese traditional medicine has accumulated rich clinical experience in the treatment of RA. HGWG has been used as a classic prescription of RA for thousands of years. Its effectiveness has already been experienced, but still needs to be scientifically confirmed with solid evidence.^[30,31]^ Therefore, we plan to carry out this multicenter randomized controlled clinical research.

To the best of our knowledge, this is a well-designed, randomized, controlled trial investigating the efficacy of the HGWG for the treatment of active RA. This study is built on our preliminary open trial with a small sample, as well as hundreds of years’ use of the HGWG in China for the treatment of RA. The findings of this research will elucidate the efficacy and safety of HGWG and provide an alternative treatment for RA. In addition, our data will benefit the clinical decision-making on active RA and possibly be incorporated into future guidelines.

### 5.1 Trial status

The trial will begin on 1st April 2019, and we will complete the recruitment on 1st April 2021.

## Author contributions

QS, QQL, and YJW supervised and coordinated the clinical trial, conceived of the study, and revised the manuscript critically for important intellectual content. YRW, YL, and ZJX are co-first authors of this manuscript, contributing equally in designing, conducting the trials, and drafting the manuscript. All authors will participate in the design of the study and performing the trial. QQL will supervise the clinical trial. YY, LL, JCM, LBX, XHG, MY, XJC, QS, and YJW are responsible for recruiting the participants. All authors have read and approved the final manuscript.

**Investigation:** Yang Yu, Li Liu, Jianchun Mao, Lianbo Xiao, Xiaohua Gu, Min Yao, Xuejun Cui, Qi Shi, Yongjun Wang.

**Methodology:** Yiru Wang, Yang Liu, Zhijie Xi, Jianchun Mao, Lianbo Xiao, Xiaohua Gu, Min Yao, Xuejun Cui, Qi Shi, Yongjun Wang, Qianqian Liang.

**Supervision:** Qianqian Liang.

**Writing – original draft:** Yiru Wang, Yang Liu, Zhijie Xi.

**Writing – review & editing:** Yiru Wang.

## References

[1] Kvien TK Uhlig T Quality of life in rheumatoid arthritis. Scand J Rheumatol 2005;34:333–41. 16234180 10.1080/03009740500327727

[2] Gabriel SE The epidemiology of rheumatoid arthritis. Rheum Dis Clin North Am 2001;27:269–81. 11396092 10.1016/s0889-857x(05)70201-5

[3] Gabriel SE Crowson CS O’Fallon WM The epidemiology of rheumatoid arthritis in Rochester, Minnesota, 1955–1985. Arthritis Rheum 1999;42:415–20. 10088762 10.1002/1529-0131(199904)42:3<415::AID-ANR4>3.0.CO;2-Z

[4] Fries JF Williams CA Morfeld D Reduction in long-term disability in patients with rheumatoid arthritis by disease-modifying antirheumatic drug-based treatment strategies. Arthritis Rheum 1996;39:616–22. 8630111 10.1002/art.1780390412

[5] Zawieja D Lymphatic biology and the microcirculation: past, present and future. Microcirculation 2005;12:141–50. 15804980 10.1080/10739680590900003

[6] Zhang GG Lee W Bausell B Variability in the traditional Chinese medicine (TCM) diagnoses and herbal prescriptions provided by three TCM practitioners for 40 patients with rheumatoid arthritis. J Altern Complement Med 2005;11:415–21. 15992224 10.1089/acm.2005.11.415

[7] Lu C Zha Q Chang A Pattern differentiation in Traditional Chinese Medicine can help define specific indications for biomedical therapy in the treatment of rheumatoid arthritis. J Altern Complement Med 2009;15:1021–5. 19757979 10.1089/acm.2009.0065

[8] Keystone EC Schiff MH Kremer JM Once-weekly administration of 50 mg etanercept in patients with active rheumatoid arthritis: results of a multicenter, randomized, double-blind, placebo-controlled trial. Arthritis Rheum 2004;50:353–63. 14872476 10.1002/art.20019

[9] Felson DT Anderson JJ Boers M American College of Rheumatology. Preliminary definition of improvement in rheumatoid arthritis. Arthritis Rheum 1995;38:727–35. 7779114 10.1002/art.1780380602

[10] Emery P Hammoudeh M FitzGerald O Sustained remission with etanercept tapering in early rheumatoid arthritis. N Engl J Med 2014;371:1781–92. 25372086 10.1056/NEJMoa1316133

[11] Felson DT Anderson JJ Boers M The American College of Rheumatology preliminary core set of disease activity measures for rheumatoid arthritis clinical trials. The committee on outcome measures in rheumatoid arthritis clinical trials. Arthritis Rheum 1993;36:729–40. 8507213 10.1002/art.1780360601

[12] Lacroix BD Karlsson MO Friberg LE Simultaneous exposure-response modeling of ACR20, ACR50, and ACR70 improvement scores in rheumatoid arthritis patients treated with certolizumab pegol. CPT Pharmacometrics Syst Pharmacol 2014;3:e143. 25353186 10.1038/psp.2014.41 PMC4474165

[13] Chung CP Thompson JL Koch GG Are American College of Rheumatology 50% response criteria superior to 20% criteria in distinguishing active aggressive treatment in rheumatoid arthritis clinical trials reported since 1997? A meta-analysis of discriminant capacities. Ann Rheum Dis 2006;65:1602–7. 16504992 10.1136/ard.2005.048975 PMC1798472

[14] Dougados M Schmidely N Le BM Evaluation of different methods used to assess disease activity in rheumatoid arthritis: analyses of abatacept clinical trial data. Ann Rheum Dis 2009;68:484–9. 19074177 10.1136/ard.2008.092577 PMC2651483

[15] van Gestel AM Prevoo ML van’t Hof MA Development and validation of the European League Against Rheumatism response criteria for rheumatoid arthritis. Comparison with the preliminary American College of Rheumatology and the World Health Organization/International League Against Rheumatism Criteria. Arthritis Rheum 1996;39:34–40. 8546736 10.1002/art.1780390105

[16] van der Heijde DM van’t Hof M van Riel PL Development of a disease activity score based on judgment in clinical practice by rheumatologists. J Rheumatol 1993;20:579–81. 8478878

[17] van Gestel AM Haagsma CJ van Riel PL Validation of rheumatoid arthritis improvement criteria that include simplified joint counts. Arthritis Rheum 1998;41:1845–50. 9778226 10.1002/1529-0131(199810)41:10<1845::AID-ART17>3.0.CO;2-K

[18] Prevoo ML van’t Hof MA Kuper HH Modified disease activity scores that include twenty-eight-joint counts. Development and validation in a prospective longitudinal study of patients with rheumatoid arthritis. Arthritis Rheum 1995;38:44–8. 7818570 10.1002/art.1780380107

[19] Bruce B Fries JF The Stanford Health Assessment Questionnaire: a review of its history, issues, progress, and documentation. J Rheumatol 2003;30:167–78. 12508408

[20] Bruce B Fries JF The Health Assessment Questionnaire (HAQ). Clin Exp Rheumatol 2005;23 (5 Suppl 39):S14–8. 16273780

[21] Pehlivan S Karadakovan A Pehlivan Y Sleep quality and factors affecting sleep in elderly patients with rheumatoid arthritis in Turkey. Turk J Med Sci 2016;46:1114–21. 27513413 10.3906/sag-1506-82

[22] Soldatos CR Dikeos DG Paparrigopoulos TJ Athens Insomnia Scale: validation of an instrument based on ICD-10 criteria. J Psychosom Res 2000;48:555–60. 11033374 10.1016/s0022-3999(00)00095-7

[23] Loge JH Kaasa S Hjermstad MJ Translation and performance of the Norwegian SF-36 Health Survey in patients with rheumatoid arthritis. I. Data quality, scaling assumptions, reliability, and construct validity. J Clin Epidemiol 1998;51:1069–76. 9817124 10.1016/s0895-4356(98)00098-5

[24] Bonovas S Minozzi S Lytras T Risk of malignancies using anti-TNF agents in rheumatoid arthritis, psoriatic arthritis, and ankylosing spondylitis: a systematic review and meta-analysis. Expert Opin Drug Saf 2016;15 (sup1):35–54. 10.1080/14740338.2016.1238458 27924644

[25] Minozzi S Bonovas S Lytras T Risk of infections using anti-TNF agents in rheumatoid arthritis, psoriatic arthritis, and ankylosing spondylitis: a systematic review and meta-analysis. Expert Opin Drug Saf 2016;15 (sup1):11–34. 27924643 10.1080/14740338.2016.1240783

[26] Bongartz T Sutton AJ Sweeting MJ Anti-TNF antibody therapy in rheumatoid arthritis and the risk of serious infections and malignancies: systematic review and meta-analysis of rare harmful effects in randomized controlled trials. JAMA 2006;295:2275–85. 16705109 10.1001/jama.295.19.2275

[27] Singh JA Hossain A Tanjong GE Biologics or tofacitinib for people with rheumatoid arthritis unsuccessfully treated with biologics: a systematic review and network meta-analysis. Cochrane Database Syst Rev 2017;3: CD012591. 10.1002/14651858.CD012591 PMC6472522 28282491

[28] Lipsky PE van der Heijde DM St CEW Infliximab and methotrexate in the treatment of rheumatoid arthritis. Anti-tumor necrosis factor trial in rheumatoid arthritis with concomitant therapy study group. N Engl J Med 2000;343:1594–602. 11096166 10.1056/NEJM200011303432202

[29] Cohen SB Emery P Greenwald MW Rituximab for rheumatoid arthritis refractory to anti-tumor necrosis factor therapy: Results of a multicenter, randomized, double-blind, placebo-controlled, phase III trial evaluating primary efficacy and safety at twenty-four weeks. Arthritis Rheum 2006;54:2793–806. 16947627 10.1002/art.22025

[30] Peng W Liang H Sibbritt D Complementary and alternative medicine use for constipation: a critical review focusing upon prevalence, type, cost, and users’ profile, perception and motivations. Int J Clin Pract 2016;70:712–22. 27354244 10.1111/ijcp.12829

[31] Jatau AI Aung MM Kamauzaman TH Use and toxicity of complementary and alternative medicines among patients visiting emergency department: Systematic review. J Intercult Ethnopharmacol 2016;5:191–7. 27104042 10.5455/jice.20160223105521 PMC4835996

